# Palliative Care in Children: The Role of a Pediatric Dentist in the Indian Scenario

**DOI:** 10.4103/0973-1075.78455

**Published:** 2011

**Authors:** K Kiran, BK Kamala

**Affiliations:** Department of Pedodontics, KLEVK Institute of Dental Sciences, Nehru Nagar, Belgaum, India; 1 The Oxford Dental College and Hospital, Bangalore, Karnataka, India

Sir,

Palliative care dentistry was defined by Wiseman[1] as the study and management of patients with active progressive and far advanced disease in whom the oral cavity has been compromised either by the disease directly or by its treatment; the focus of care is quality of life. Palliative care for children aims to improve the quality of life of the pediatric patient as well as their family. This is done through expert management of pain and other symptoms. It is also done through physical, social, emotional, and spiritual support services, offering the patient and family specialized counseling to help them cope with the result from dealing with a serious illness or condition. The pediatric dentist may be the first person to diagnose such conditions in children. According to the American Academy of Paediatric Dentistry (AAPD), pediatric dentistry is defined as an age defined specialty that provides both primary and comprehensive preventive and therapeutic oral health care for infants and children through adolescence, including those with special health care needs. A pediatric dentist has a significant role in enhancing the oral health integrity of the children affected with life-threatening illnesses. In India, the role of a dental professional[2] in palliative care is in its infancy.

The American Academy of Paediatrics (AAP),[3] supports an integrated model of palliative care “in which the components of palliative care are offered at diagnosis and continued throughout the course of illness, whether the outcome ends in cure or death”. Reserving palliative care for children who have exhausted every curative treatment and are dying would mean that many other children would miss out on the benefits that palliative care can offer. Including children who have a life-threatening illness or condition but are still receiving curative treatment ensures that all children who can benefit from palliative care have access to it. The AAP recommends the development and broad availability of pediatric palliative care services based on child-specific guidelines and standards. Such services will require widely distributed and effective palliative care education of pediatric health care professionals.

The past two decades have seen palpable changes in the mindset of health care providers, and policy makers with respect to the urgency of providing palliative care.[4] Even the most severely affected children can retain a certain degree of oral health by using conscious sedation techniques and general anesthesia. With aggressive preventive interventions and management of oral diseases, a pediatric dentist can bring about a positive change in alleviating the pain, discomfort, and sufferings of these children.

Developing and promoting all the eras of palliative care in a community setup is a must for the proper palliative care of the needy. Palliative care is best provided using an integrated multidisciplinary approach.[3,5] There is a need to increase the awareness among the community with regard to a child’s oral health in palliative care and its role at the end stage of any chronic illness. Greater awareness of palliative care should make it easier for professionals to combine active treatment and a supportive approach. People with disabling, progressive illnesses expect active care, but they also seek comfort, control, and dignity. The barriers to effective communication about emotional and end-of-life issues are well recognized.[6] The pediatric dentists should be trained to participate actively in administering palliative therapy to children. Levy-Polack *et al.*[7] showed that the systematic application of a preventive protocol significantly reduces the incidence of oral complications. The results of this study identify a need to include a pediatric dentist in a multidisciplinary team which provides oral care for cancer patients.

In India, a system has to be put in place that allows the caregivers to assess children with progressive, debilitating conditions including a process for timely referral to the pediatric dentist to realize the goal of improving the overall health of the child.
